# Acquired hemophilia A and plasma cell neoplasms: a case report and review of the literature 

**DOI:** 10.1186/s13256-020-02505-7

**Published:** 2020-10-30

**Authors:** Katarzyna A. Jalowiec, Martin Andres, Behrouz Mansouri Taleghani, Albulena Musa, Martina Dickenmann, Anne Angelillo-Scherrer, Alicia Rovó, Johanna A. Kremer Hovinga

**Affiliations:** Department of Hematology and Central Hematology Laboratory, Inselspital, Bern University Hospital, University of Bern, CH-3010 Bern, Switzerland

**Keywords:** Acquired hemophilia A, Multiple myeloma, Soldering multiple myeloma, Plasma cell diseases, Bleeding diathesis

## Abstract

**Background:**

Acquired hemophilia A is a rare autoimmune disease with clinically often significant bleeding diathesis resulting from circulating autoantibodies inhibiting coagulation factor VIII. Half of acquired hemophilia A cases are associated with an underlying disorder, such as autoimmune diseases, cancer, or use of certain drugs, or occur during pregnancy and in the postpartum period. In the other half, no underlying cause is identified. An association of acquired hemophilia A with plasma cell neoplasm seems to be extremely rare.

**Case presentation:**

We describe a case of a 77-year-old Swiss Caucasian man who was diagnosed with acquired hemophilia A and smoldering multiple myeloma as an underlying cause. Acquired hemophilia A was treated with prednisolone, cyclophosphamide, and immunoadsorption. Extensive workup revealed a plasma cell neoplasm as the only disorder associated with or underlying the acquired hemophilia A. For long-term control of acquired hemophilia A, we considered treatment of the plasma cell neoplasm necessary, and a VRD (bortezomib, lenalidomide, and dexamethasone) regimen was initiated. Due to multiple complications, VRD was reduced to VRD-lite after two cycles. After nine cycles of induction therapy and five cycles of consolidation therapy, the patient is in complete remission of his acquired hemophilia A and very good partial remission of the plasma cell neoplasm. We conducted a literature review to identify additional cases of this rare association and identified 15 other cases. Case descriptions, including the sequence of occurrence of acquired hemophilia A and plasma cell neoplasm , treatment, evolution, and outcome are presented.

**Discussion and conclusions:**

Our case, together with 15 other cases described in the literature, underscore the possibility of plasma cell neoplasm as an underlying cause of acquired hemophilia A. Physicians should consider including protein electrophoresis, immunofixation, and analysis of free light chains in laboratory diagnostics when treating a patient with acquired hemophilia A. The occurrence of excessive and unexplained bleeding in patients diagnosed with plasma cell neoplasm should raise suspicion of secondary acquired hemophilia A and trigger the request for coagulation tests, particularly in patients treated with immunomodulatory drugs such as thalidomide or lenalidomide. Additionally, early intervention with immunoadsorption can be lifesaving in cases with high-titer factor VIII inhibitors, especially when surgical interventions are necessary.

## Background

Acquired hemophilia A (AHA) is a rare autoimmune disease with a clinically significant bleeding diathesis, resulting from circulating autoantibodies inhibiting coagulation factor VIII (FVIII). The incidence is estimated to be 1–1.5 cases per 1 million population, and AHA is most often encountered in elderly patients. Half of AHA cases are associated with an underlying disorder, such as autoimmune diseases, cancer, use of certain drugs, or occur during pregnancy, and in the postpartum period. In the other half, no underlying cause is identified (idiopathic AHA). Typically, bleeding is located in the skin, mucosa, or soft tissues, and, in contrast to congenital hemophilia, joint bleeding is rare. Mortality in AHA is increased, particularly in elderly patients and in patients with underlying malignancies. Despite frequent bleeding complications and complications of immunosuppressive therapy, the primary cause of death in AHA is the underlying disease [1].

Management of AHA is based on four pillars: (1) avoidance of procedures that may induce bleeding, (2) control of bleeding, (3) inhibitor eradication, and (4) treatment of the underlying disease. The mainstays of bleeding management are bypassing agents, such as activated prothrombin complex concentrates (aPCCs) and recombinant activated factor VII (rFVIIa). More recently, recombinant porcine FVIII, which lacks complete sequence homology with human FVIII, has become available. Different strategies, such as immunosuppression with steroids alone or in combination with cyclophosphamide, immunoglobulins, rituximab, plasma exchange, and immunoadsorption, are in use for inhibitor elimination and eradication and, with that, restoration of FVIII clotting activity [1].

Hematological malignancies may be associated with or may be the underlying cause of AHA. Among these malignancies, lymphoproliferative disorders are most common. An association of AHA with plasma cell neoplasm (PCN) seems to be extremely rare and to represent only 14% of AHA cases associated with a hematological malignancy [2]. We describe a case of a 77-year-old man who presented with AHA and smoldering multiple myeloma as an underlying cause.

## Case presentation

**Fig. 1:**
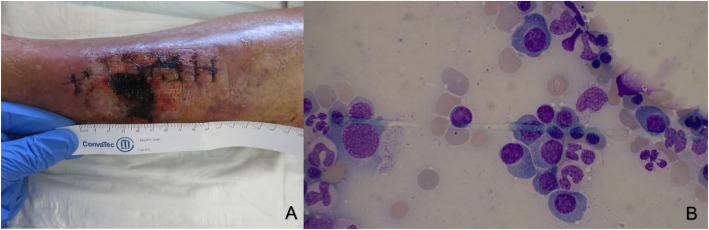
Findings in our patient. **a** Presentation of compartment syndrome following surgery. **b** Plasma cells on bone marrow aspirate

A 77-year-old Swiss Caucasian man was admitted to a peripheral hospital due to a compartment syndrome of his left calf following a minor trauma (Fig. 1a). A large (13 × 8 × 0.6 cm) isolated calf hematoma was documented on ultrasound, but the patient’s further physical evaluation was unremarkable. At admission, anticoagulant therapy with apixaban, prescribed for atrial fibrillation, was interrupted. The patient’s family and personal history were negative for hematological diseases. Two months earlier, the patient had undergone an uneventful colonoscopy with polypectomy initiated because of melena. No coagulation tests were available from that time. Despite two surgical interventions, the bleeding into the calf persisted. Six days after initial admission, AHA was suspected, and the patient was transferred to our hospital.

The results of the patient’s laboratory evaluation revealed a prolonged activated partial thromboplastin time (aPTT; 119 s; reference range 25.0–36.0 s), and FVIII:C of 2% in the presence of a high-titer FVIII inhibitor of 102 Bethesda units (BU)/ml confirmed the diagnosis of AHA. Additional diagnostic workup revealed an IgG kappa paraprotein of 9.2 g/L as well as a slightly reduced hemoglobin of 106 g/L. The patient’s albumin corrected serum calcium was 2.38 mmol/L, serum creatinine 103 μmol/L, β_2_-microglobulin 3.0 mg/L, lactate dehydrogenase 301 U/L, free light chains kappa 41.1 mg/L and lambda 10.9 mg/L, and free light chain ratio of 3.77. Magnetic resonance imaging excluded multiple myeloma defining focal bone lesions. A subsequent bone marrow biopsy showed infiltration of clonal plasma cells of 15% (Fig. 1b). Conventional cytogenetic analysis was not performed; however, microarray-based comparative genomic hybridization revealed hyperdiploidy with trisomy 3, 4, 5, 9, 11, and 21, whereas the result of fluorescence in situ hybridization was negative for MYC, IGH rearrangement, and 17p. Apart from a smoldering myeloma, no other diseases potentially underlying the AHA were identified.

Treatment with prednisolone (1 mg/kg body weight) and cyclophosphamide (150 mg/day) was started the day of presentation at our hospital. Because of imminent compartment syndrome and potential need of further surgical interventions, immunoadsorption was started according to the modified Bonn Malmö protocol [3] to rapidly deplete the FVIII inhibitor. Altogether, we performed seven immunoadsorption sessions processing approximately two total patient plasma volumes per session over the next 13 days. Within 1 month, aPTT and FVIII:C had normalized, whereas the inhibitor titer had significantly decreased but was still detectable (1.04 BU/ml).

Insertion of a central venous catheter for immunoadsorption, as well as the bone marrow biopsy, were done under substitution with rFVIIa (NovoSeven; Novo Nordisk, Plainsboro, NJ, USA). One to three doses of 90 μg/kg body weight were applied. Wound healing at the calf was delayed, and due to skin necrosis, surgical revision became necessary. At that time, the patient’s aPTT had already normalized, and substitution with rFVIIa was not needed.

Lack of complete remission of AHA prompted us to initiate a VRD (bortezomib, lenalidomide, dexamethasone) regimen to treat the patient’s smoldering myeloma. After two cycles, the FVIII inhibitor had further declined to 0.31 BU/ml. The monoclonal immunoglobulin was still detectable by immune fixation but no longer quantifiable. Subsequently, treatment intensity was reduced to a VRD-lite regimen [4] because of thrombocytopenia, mild polyneuropathy, and signs of congestive heart failure. During follow-up, no further bleeding occurred. After nine cycles of induction therapy and five cycles of consolidation therapy with the VRD-lite regimen, the patient is in complete remission of his AHA and in very good partial remission of his PCN (Table 1, patient 16).

**Table 1:** Characteristics of patients with association of AHA and plasma cell neoplasms – a systematic review

Patient no.	Author, year [reference]	Sex	Age (years)	Diagnosed first	Bleeding	FVIII:C (%)	FVIII inhibitor (BU/ml)	Hemostatic treatment	Treatment AHA inhibitor eradication	AHA outcome	Paraprotein	Treatment PCN	PCN outcome	Alive/died
1	Glueck *et al*., 1965 [5]	M	70	PCN	MC, RT	NA	NA	NA	C	NA	NA	NA	NA	NA
2	Loftus *et al*., 1994 [6]	F	58	PCN	MC, A	8	36	FVIII, pFVIII	S, C	Bleeding continued	Lambda light chain	M	NA	Died of intra-abdominal bleeding
3	Stricker *et al*., 1994^a^ [7]	M	52	PCN	I	2	17.8	FVIII, plasma	S, PEX	Normal APTT and FVIII:C	Kappa light chain	IFN-α, ASCT	CR	Died of sudden cardiac death
4	Sallah *et al*., 2000 [8]	F	58	AHA	MC	< 1	28	pFVIII, APCC	S, PEX	Inhibitor persisted	NA	M	Died	Died of acute renal failure/hemorrhage
5	Holme *et al*., 2005 [9]	M	58	AHA	I	6	20	APCC	S, C	NA	NA	NA	PR	Alive
6	Sari *et al*. 2009 [10]	F	43	AHA	I	6	10	No treatment	No treatment	Normal coagulation	IgG kappa	VinOD, ASCT	CR	NA
7	Decaux *et al*. 2009 [11]	F	44	PCN	MC	6	29	rFVIIa	R	NA	IgA kappa	NA	NA	NA
8	Muzaffar *et al*. [12]	M	65	PCN	PE, HA	< 5	9.5	APCC, plasma	IVIG, R	FVIII:C 22%, no FVIII inhibitor	Lambda light chain	VTD	CR	Alive
9	Saburi *et al*. 2015^a^ [13]	F	67	PCN	NA	2	4.9	No treatment	S, C	Normal APTT, FVIII inhibitor 4.85 BU/ml	NA	VD, LCD	CR	NA
10	Ross *et al*. [14]	F	64	AHA	MC, HP	17	5	rFVIIa	S	Normal coagulation	IgM kappa	VTD	NA	NA
11	Innao *et al*. [15]	M	67	PCN	NA	28	NA	FVIII	No treatment	Normal coagulation	IgG kappa, kappa light chain	VMP, ASCT	CR	Alive
12	Brás, et al. [16]	M	87	PCN	MC, IM	1.4	18.4	APPC	S, C, B	Normal APTT, FVIII:C 36%, FVIII inhibitor 0.8 BU/ml	IgG kappa	MTP, VD	PR	NA
13	Napolitano *et al*. 2017 [17]	F	59	AHA	MC, HA, A	12	70	rFVIIa, APCC	S, R	Normal coagulation	IgG lambda	VMP	CR	Alive
14	Kawashima *et al*. 2018 [18]	M	52	PCN	IM	17	1	rFVIIa	No treatment	Normal coagulation	IgA kappa	VD, VCD, VTD, LD, ASCT, allo-HCT	CR	NA
15	Sourdeau *et al*. 2019 [19]	M	78	PCN	ST	< 1	19	NA	NA	NA	NA	VCD	NA	NA
16	Our patient	M	77	AHA	IM	2	102	rFVIIa^b^	S, C, IA	Normal APTT, normal FVIII	IgG kappa	VRD, RD	VGPR	Alive

*Abbreviation*: *AHA* acquired hemophilia, *PCN* plasma cell neoplasm, *NA* not available, *n.d.* not done

Bleeding: *MC* mucocutaneous bleeding (epistaxis, gingiva, soft tissue, gastro-intestinal, gynecological), *I* iatrogenic (postoperative, after biopsy or dental procedure), *A* intra-abdominal, *HA* hemarthrosis, *PE* pericardial bleeding, *HP* hemoptysis, *RT* retinal bleeding, *IM* intramuscular

Hemostatic treatment: *rFVIIa* recombinant activated factor VII, *aPCC* activated prothrombin complex concentrate, *FVIII* factor VIII (human plasma or recombinant), *pFVIII* porcine factor VIII, *plasma* fresh frozen plasma or cryoprecipitate

Other treatment: *allo-HCT* allogenic stem cell transplantation, *ASCT* autologous stem cell transplantation, *C* cyclophosphamide, *CR* complete remission, *D* dexamethasone, *IA* immunoadsorption, *INF-a* interferon alpha, *IVIG* intravenous immunoglobulin, *L* lenalidomide, *M* melphalan, *O* doxorubicin, *P* prednisone, *PEX* plasma exchange, *PR* partial remission, *R* Rituximab, *S* steroids, *T* thalidomide, *V* bortezomib, *Vin* vincristine, *VGPR* very good partial remission

^a^AHA considered a side effect of plasma cell disease treatment (discussed in text)

^b^Hemostatic treatment only for interventions (bone marrow biopsy, surgery)

## Discussion and conclusions

To further elucidate this rare association of AHA and PCN, we reviewed the published literature in PubMed using the following search terms: “hemophilia,” “inhibitor,” “factor VIII,” “myeloma,” “plasma cell disorder” or “neoplasm,” “smoldering myeloma,” “MGUS,” “monoclonal gammopathy,” and “paraprotein.” Our search identified 15 further cases. Case descriptions, including the sequence of occurrence of AHA and PCN, treatment, evolution, and outcome, are provided in Table 1.

We found nine male and seven female patients diagnosed with AHA and PCN. Their median age at diagnosis of AHA was 61.5 (range 43–87) years. Soft tissue bleeding was the most common type of bleeding (7 of 16 patients; 43%), in line with other reports [1]. The patients’ median FVIII inhibitor titer was 18.7 BU/ml (range 1–102 BU/ml; no data available for two patients). AHA was diagnosed after excessive postintervention hemorrhage in two patients and in one patient following life-threatening pericardial bleeding and hemarthrosis. AHA with active bleeding was the presenting sign and preceded PCN diagnosis in six cases (38%) (Table 1, cases 4, 5, 6, 10, 13, and 16), whereas in the other cases, PCN was diagnosed first. In three of the latter cases, AHA was considered to have occurred secondary to multiple myeloma treatment. The implicated drugs were interferon alpha, lenalidomide, and thalidomide. Information on the type of paraprotein was available in 11 cases, but no particular immunoglobulin type or clonal light-chain was discernible.

Hemostatic treatment with bypassing agents was necessary in 11 cases. Their median FVIII inhibitor titer was 19.2 (range 1–70) BU/ml, whereas the three patients who did not need hemostatic treatment had FVIII inhibitor titers of 4.85, 10, and 102 BU/ml, respectively. This underscores the fact that FVIII inhibitor titers in AHA do not necessarily correlate with the severity of the bleeding manifestations. Our patient received rFVIIa only prophylactically before catheter insertion and bone marrow biopsy. Under immunoadsorption, a rapid increase of FVIII clotting activity reaching safe levels was observed, and further treatment with bypassing products was not necessary.

Inhibitor eradication was attempted with steroids alone (n = 4) or in combination with cyclophosphamide (n = 5), with cyclophosphamide alone (n = 1), and with rituximab (n = 3). Plasma exchange and immunoadsorption to remove FVIII antibodies were performed in two and one patient (our patient), respectively. Given the long observation period of 55 years, different therapy regimens were used to treat the underlying PCN in the 16 cases (details are given in Table 1). At the time of reporting, 13 patients were alive, whereas two of the three patients who died, died of bleeding complications. Information on the outcome of AHA and/or PCN was available for nine of 13 survivors: six had normal coagulation tests, and FVIII activity was mildly reduced in two. PCN was in complete or partial remission in seven and two cases, respectively. One patient (case 7 in Table 1) had a spontaneous remission of his FVIII inhibitor without any treatment.

In summary, our patient’s case, together with the 15 other cases described in the literature, underscores the possibility of PCN as an underlying cause of AHA. Serum and urine protein electrophoresis is not (everywhere) part of standard workup of AHA, and underreporting of this association is possible or even likely. Accordingly, physicians should consider including protein electrophoresis, immunofixation, and analysis of free light chains in laboratory diagnostics when treating a patient with AHA. The occurrence of excessive and unexplained bleeding in patients diagnosed with PCN should raise the suspicion of secondary AHA and trigger the request for coagulation tests, particularly in patients treated with immunomodulatory drugs such as thalidomide or lenalidomide. Whether PCN treatment alone can control AHA in these cases remains an open question; 11 of 16 (69%) of the reported cases received treatment for both diseases.

In our experience, early intervention with immunoadsorption can be lifesaving in cases with high FVIII inhibitor titers, especially in patients requiring a surgical intervention. The modified Bonn Malmö protocol [3] is useful to guide AHA therapy that includes immunoadsorption.

## Data Availability

Not applicable. All data are included in the article.

## Abbreviations

A: Intra-abdominal
AHA: Acquired hemophilia
aPCC: Activated prothrombin complex concentrate
ASCT: Autologous stem cell transplant
C: Cyclophosphamide
CR: Complete remission
D: Dexamethasone
FVIII: Factor VIII
HA: Hemarthrosis
HCT: Allogeneic stem cell transplant
HP: Hemoptysis
I: Iatrogenic
IA: Immunoadsorption
IM: Intramuscular
IFN: Interferon
IVIG: Intravenous immunoglobulin
L: Lenalidomide
M: Melphalan
MC: Mucocutaneous bleeding
n.d.: Not done
NA: Not available
O: Doxorubicin
P: prednisone
PCN: Plasma cell neoplasm
PE: Pericardial bleeding
PEX: Plasma exchange
pFVIII: Porcine factor VIII
PR: Partial remission
R: Rituximab
rFVIIa: Recombinant activated factor VII
RT: Retinal bleeding
S: Steroids
T: Thalidomide
V: Bortezomib
VGPR: Very good partial remission
Vin: Vincristine
